# Relationship between Th17-mediated immunity and airway inflammation in childhood neutrophilic asthma

**DOI:** 10.1186/s13223-020-00504-3

**Published:** 2021-01-06

**Authors:** Qing Wei, Jing Liao, Min Jiang, Jing Liu, Xiuan Liang, Guangmin Nong

**Affiliations:** Department of Pediatrics, The First Affiliated Hospital of Guangxi Medical University, Nanning, 530021 Guangxi China

**Keywords:** Asthma, Neutrophils, Th17 cells, IL-17, Children

## Abstract

**Background:**

The pathogenetic mechanisms of neutrophilic asthma are not well understood now. Whether T helper (Th)17-mediated immunity contributes to the pathogenesis of neutrophilic asthma in human is still under investigation. The aim of this study was to explore the relationship between Th17-mediated immunity and airway inflammation in childhood neutrophilic asthma.

**Methods:**

Twenty-eight children with exacerbated asthma and without using any glucocorticoids were divided into three groups: eosinophilic asthma (EA, n = 12) group, neutrophilic asthma (NA, n = 10) group and paucigranulocytic asthma (PGA, n = 6) group according to the induced sputum cytology. Ten healthy children were recruited as healthy control (HC, n = 10) group. Peripheral Th17 and Th2 cells, and the expression of Ki-67 in peripheral Th17 cells were detected by flow cytometry. The mRNA expression of retinoic acid-related orphan receptor γt (RORγt) in peripheral blood mononuclear cells (PBMCs) was detected by qRT-PCR. The concentrations of IL-17, IL-8 and IL-5 in sputum, as well as IL-17 in plasma and culture supernatant of activated PBMCs were measured by ELISA.

**Results:**

The percentage of Th17 cells in peripheral Th cells, and the concentrations of IL-17, IL-8 in sputum, as well as IL-17 in culture supernatant of activated PBMCs were all increased in NA group, and positively correlated with neutrophil level in sputum and with each other. Also, the mRNA expression of RORγt in PBMCs and Ki-67 positivity in peripheral Th17 cells were both increased in NA group. The percentage of Th2 cells in peripheral Th cells, and the concentration of IL-5 in sputum were both increased in EA group, and positively correlated with eosinophil level in sputum and with each other.

**Conclusions:**

Both Th17- and Th2-mediated immunity are involved in the pathogenesis of childhood asthma. There is predominance of Th17-mediated immunity and Th17 cells proliferation in childhood neutrophilic asthma.

## Background

Bronchial asthma (Asthma) is one of the most common chronic airway diseases worldwide. It’s well know that airway inflammation plays a key role in the pathogenesis of asthma. According to the phenotype of airway inflammation, asthma can be classified into different subtypes: eosinophilic asthma (EA) and non-eosinophilic asthma (NEA) [1]. Neutrophilic asthma (NA) is a common subtype of NEA, and is characterized by the mass infiltration of neutrophilic rather than eosinophilic airway inflammation [1]. NA occurs in 15–25% of the asthmatics, and is more prevalent among the steroid-dependent severe asthmatics [2, 3]. Many cells (such as eosinophils, mast cells, T lymphocytes, neutrophils, and airway epithelial cells, etc.) and cellular elements participate in the pathogenesis of airway inflammation in asthma, in which T lymphocytes play a critical role [4–8]. Typically, it is known that T helper (Th)2 cells can secrete interleukins (IL-4, IL-5 and IL-13), and promote eosinophilic airway inflammation in atopic asthma [7–9]. Whereas, it has been shown that the eosinophilic/atopic phenotype of asthma driven by Th2 mechanisms is not the only immunologic pathway contributing to asthma [7, 8]. Recently, a new T helper cell subset has been identified, namely, Th17 cells, which is characterized by the secretion of IL-17 and the expression of retinoic acid-related orphan receptor γt (RORγt), and can promote neutrophilic inflammation [10]. Some studies, especially in the murine asthmatic models have showed that Th17 cells and IL-17 also seemed to be involved in asthma pathogenesis [11, 12]. Whereas, whether Th17-mediated immunity contributes to the pathogenesis of neutrophilic asthma in human remains to be investigated. The purpose of this study was to investigate the relationship between Th17-mediated immunity and airway inflammation in childhood with neutrophilic asthma.

## Materials and methods

### Subjects

Twenty-eight children with exacerbated asthma and without using any glucocorticoids were recruited from the pediatric department of the First Affiliated Hospital of Guangxi Medical University (Nanning, Guangxi, China). The subjects had no respiratory infection in the past 4 weeks. The diagnosis and severity assessment of asthma were performed according to Global Initiative for Asthma (GINA) guidelines [13]. Spirometry (MasterScreen, JAEGER, Germany) was performed as recommended by the GINA guidelines [13]. Ten healthy children with no respiratory symptoms and normal lung function were recruited as the healthy control group (HC group) (n = 10). All the participants were given informed consent prior to their inclusion in the study. The study was approved by the institutional ethics review board of the First Affiliated Hospital of Guangxi Medical University.

### Sputum induction and processing

Sputum was induced using hypertonic saline (4.5% for mild-moderate asthma and controls; 0.9% for severe asthma) delivered via an ultrasonic nebuliser as previously described [14]. Induced sputum was selected from saliva, and processed within 2 h for the preparation of cell suspension by adding four volumes of phosphate-buffered saline solution (PBS) and mixed by rotating for 15 min at 22 °C. Then, the cell suspension was centrifuged at 400 g for 10 min. The supernatant was aspirated and stored at − 80 °C for cytokine assays, and the cell pellet was resuspended by adding a volume of 0.1% dithiothreitol and mixed by rotating for 15 min at 22 °C. The suspension was then filtered, and a total cell count and viability were determined. The remaining sample was centrifuged and the cell pellet was diluted with PBS to 1 × 10^6^ cells/mL. Slides were prepared by using cytospin (Cytopro 7620,Wescor, USA) and stained with Diff-Quick stain for differential cell counts, 400 nonsquamous cells were counted. Results were expressed as a percentage of total non-squamous cells.

### Classification of asthma phenotypes

Based on previous studies [15, 16], subjects with ≥ 2% sputum eosinophils and < 61% sputum neutrophils were classified as eosinophilic asthma (EA), and those with > 61% sputum neutrophils and < 2% sputum eosinophils were classified as neutrophilic asthma (NA). Subjects with ≤ 61% sputum neutrophils and < 2% sputum eosinophils were classified as paucigranulocytic asthma (PGA). Twenty-eight children with exacerbated asthma in this study were divided into three groups: EA group (n = 12), NA group (n = 10) and PGA group (n = 6) according to the induced sputum cytology.

### Flow cytometry

Heparinized blood samples from asthmatic patients and healthy controls were obtained and erythrocytes were lysed by BD Pharm Lyse™ Lysing Buffer (BD Biosciences, USA). The cells (1 × 10^6^ cells/ml) were thereafter incubated in 24-well cell culture plates in RPMI 1640 cell culture medium (Gibco, USA) containing 10% heat-inactivated FBS, 2 mM l-glutamine, 20 mM HEPES, 100 U/ml penicillin, 100 µg/ml streptomycin, and activated with phorbol-12-myristate-13-acetate (PMA) (25 ng/ml) (Sigma, USA) and ionomycin calcium salt (1 μg/ml) (Sigma) for 1 h at 37 °C in a humidified 5% CO_2_ atmosphere, then stimulated for additional 4 h in the presence of protein transport inhibitor (0.7 μl/ml) (BD GolgiStop™, BD Biosciences, USA). Th17, Th2 cells and nuclear-associated antigen Ki-67expressed in Th17 cells were detected by surface antigen and intracellular cytokine staining using flow cytometry. The cells were washed in ice-cold PBS followed by staining with APC-labeled anti-human CD4 (BD Biosciences) and PerCP-Cy™ 5.5-labeled anti- human CD8 (BD Biosciences) for 30 min at 4 °C in the dark. Samples were washed twice in PBS before fixation and permeabilization using Transcription Factor Buffer Set (BD Biosciences) as recommended by the manufacturer. Intracellular staining was performed at 4 °C for 50 min in the dark with PE-labeled anti-human cytokine antibodies (IL17, IL4, BD Biosciences) and Alexa Fluor 488-labeled anti-human cytokine antibodies (Ki-67, BD Biosciences). PE-conjugated Mouse IgG1κ and Alexa Fluor 488-conjugated Mouse IgG1κ (BD Biosciences) were used as isotype controls. Four-color flow cytometry was performed by a FACS Calibur System (Becton–Dickinson, USA). Results were analyzed by FCS Express 4 software (De Novo Software, Canada).

### Peripheral blood mononuclear cell (PBMC) culture and stimulation

Blood samples from patients and healthy controls were collected in acid citrate dextrose vacutainer tubes (Becton–Dickinson, USA) and used for plasma selection and PBMC isolation. The cells were isolated by density gradient centrifugation on the lymphocyte separation medium Lymphoprep with a density of 1.077 g/ml, PBMCs were collected and washed twice with PBS. The cells (1 × 10^6^ cells/ml) were cultured for 5 h in 24-well cell culture plates in complete medium in presence of PMA (25 ng/ml) (Sigma) and ionomycin calcium salt (1 μg/ml) (Sigma) at 37 °C in a humidified 5% CO_2_ atmosphere. The culture supernatant and plasma were collected and stored at − 80 °C for cytokine assays. The cells were processed for real-time quantitative reverse transcription-polymerase chain reaction (qRT-PCR) analysis.

### qRT-PCR

Total RNA was extracted from PBMCs using a E.Z.N.A.^®^ Total RNA Kit I (OMEGA, USA), then the cDNAs were synthesized using a PrimeScript™ RT reagent Kit (TaKaRa, RR037S, Dalian, China), following the instructions provided by the manufacturer. Based on mRNA sequences of RORγt in Genbank, the primers were designed with primer premier 5.0 software and synthesized by Sangon Biotech Co.,Ltd (Shanghai, China). The following primers were used: RORγt, 5′-CCTGGGCTCCTCGCCTGACC-3′ (forward primer) and 5′-TCTCTCTGCCCTCAGCCTTGCC-3′ (reverse primer) with the amplicon size of 169 bp, β-actin, 5′-CACGAAACTACCTTCAACTCC-3′ (forward primer) and 5′-CATACTCCTGCTTGCTGATC-3′ (reverse primer) with the amplicon size of 262 bp. The qRT-PCR was performed with a 7500 Real Time PCR system (Applied Biosystems, USA) using FastStart Universal SYBR Green Master (ROX) (Roche, Swiss) in a 20 µl reaction volume containing 200 nM primers and 5 ng cDNA. Thermal cycling was initiated with a 10-min denaturation at 95 °C, followed by 40 cycles of 95 °C for 15 s and 60 °C for 60 s. The relative RORγt mRNA expressions were normalized to the level of β-actin (the housekeeping gene) transcripts and quantified by the 2^−∆∆Ct^ method using 7500 System Sequence Detection software (Applied Biosystems).

### Cytokine analysis

The levels of IL-17A in sputum supernatant, culture supernatant from PMA-stimulated PBMCs as well as plasma, and the concentrations of IL-8, IL-5 in sputum supernatant were measured using commercial enzyme-linked immunosorbent assay (ELISA) kit (Boster, China) according to the manufacturer’s instructions. The lower detection limits were 1 pg/ml for IL-17A, 1 pg/ml for IL-8 and 1 pg/ml for IL-5, respectively.

### Statistical analysis

Statistical Package for Social Sciences version 16.0 software was used for data analysis. Normal distribution data are summarized as mean and standard deviation (SD), and those of non-normal distribution are summarized as median and interquartile range (IQR). Differences between groups were determined using one-way analysis of variance (ANOVA) with Student–Newman–Keuls post hoc test for parametric data or Kruskall Wallis test for nonparametric data. Associations between variables were examined using Pearson’s correlation coefficients. A *p* value of < 0.05 (two-tailed) was considered statistically significant.

## Results

### Demographic and clinical characteristics

Subjects’ clinical characteristics and their sputum neutrophil and eosinophil counts are shown in Table 1. Forced expiratory volume in 1 s (FEV_1_) and peak expiratory flow (PEF) were significantly lower in the NA group than those in the EA, PGA and HC groups (all *p *< 0.01).

**Table 1:** Demographic and clinical characteristics of the subjects

	HC (*n *=10)	PGA (*n *=6)	EA (*n *=12)	NA (*n *=10)
Age (years)	10.40 ± 2.42	9.08 ± 1.74	9.54 ± 2.01	9.70 ± 2.50
Gender (male: female)	6:4	3:3	7:5	6:4
FEV_1_ (% of predicted)	87.72 ± 5.32	84.90 ± 13.23	85.70 ± 12.74	68.81 ± 9.99**
PEF(% of predicted)	92.56 ± 6.28	86.73 ± 14.22	88.37 ± 11.51	70.56 ± 9.48**
Sputum neutrophils (% of total inflammatory cells)	32.57 ± 6.74	38.45 ± 9.35	40.28 ± 6.97	69.57 ± 6.80**
Sputum eosinophils (% of total inflammatory cells)	1.31 ± 0.59	1.66 ± 0.32	3.06 ± 0.40^#^	1.76 ± 0.19

Data are expressed as mean and SD

*EA* eosinophilic asthma, *FEV*_*1*_ forced expiratory volume in 1 s, *HC* healthy controls, *NA* neutrophilic asthma, *PEF* peak expiratory flow, *PGA* paucigranulocytic asthma

** *p *< 0.01 versus PGA, EA and HC. ^#^ *p *< 0.01 versus PGA, NA and HC

### Levels of Th17 and Th2 cells in peripheral blood

**Fig. 1:**
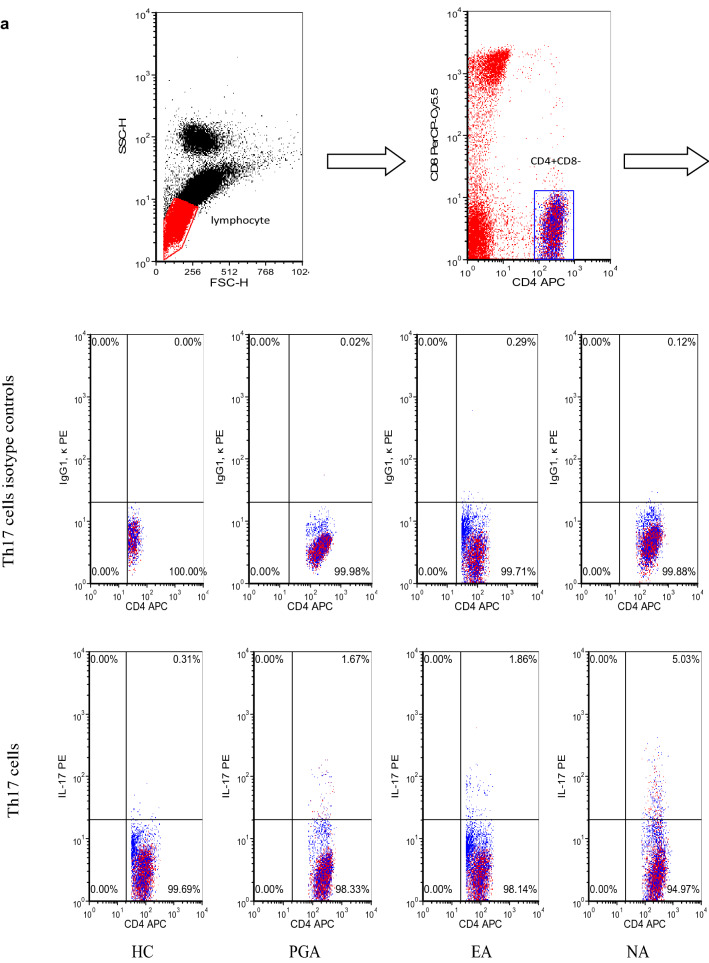

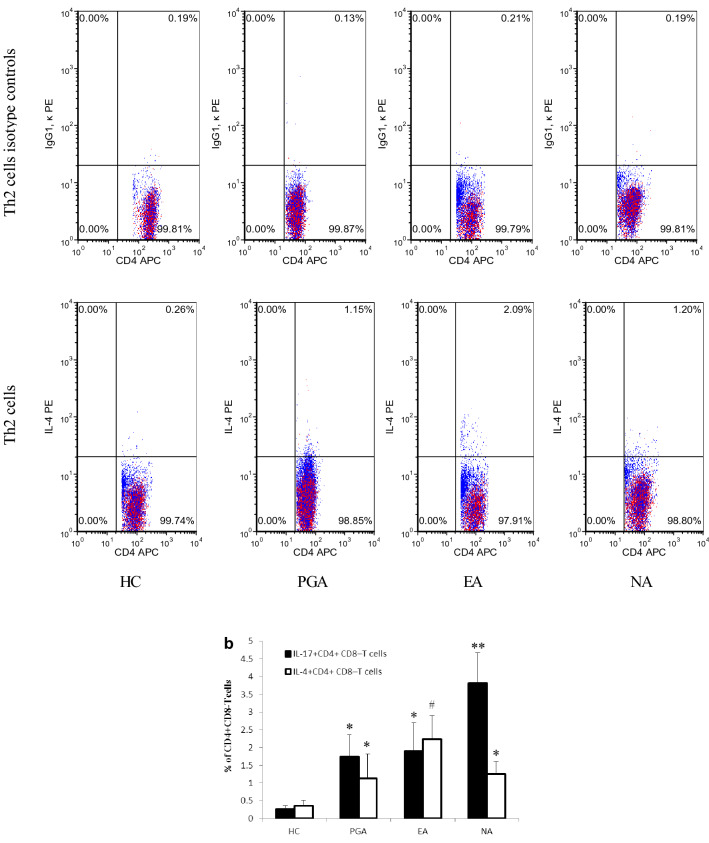
**a** Representative flow cytometric profiles of IL-17^+^CD4^+^ CD8^−^ T cells and IL-4^+^CD4^+^ CD8^−^ T cells from asthmatics and healthy control. **b** Percentage of IL-17^+^CD4^+^ CD8^−^ T cells and IL-4^+^CD4^+^ CD8^−^ T cells in CD4^+^ CD8^−^ T cells from asthmatics and healthy controls. *p < 0.01 versus healthy controls. **p < 0.01 versus PGA, EA and HC. ^#^*p *< 0.01 versus PGA, NA and HC. *EA* eosinophilic asthma, *HC* healthy controls, *NA* neutrophilic asthma, *PGA* paucigranulocytic asthma

Peripheral blood cells from asthmatic patients and healthy controls were stimulated with PMA and ionomycin. The expression of surface antigens and intracellular cytokines was measured by flow cytometry. CD4^+^CD8^−^ cells were defined as T helper (Th) cells, IL-17^+^CD4^+^ CD8^−^ T cells were defined as Th17 cells and IL-4^+^CD4^+^ CD8^−^ T cells were defined as Th2 cells. Representative flow cytometric profiles of IL-17^+^CD4^+^ CD8^−^ T cells and IL-4^+^CD4^+^ CD8^−^ T cells from the samples are shown in Fig. 1a. Percentages of Th17 and Th2 cells in peripheral Th cells were significantly higher in asthmatics compared to those in healthy controls (*p *< 0.01), in which the percentage of Th17 cells in peripharal Th cells was significantly higher in the NA group than those in the EA and PGA groups (*p *< 0.01), whereas the percentage of Th2 cells in peripheral Th cells was significantly higher in the EA group than those in the NA and PGA groups (*p *< 0.01) (Fig. 1b).

### Expression of Ki-67 in Th17 cells

**Fig. 2:**
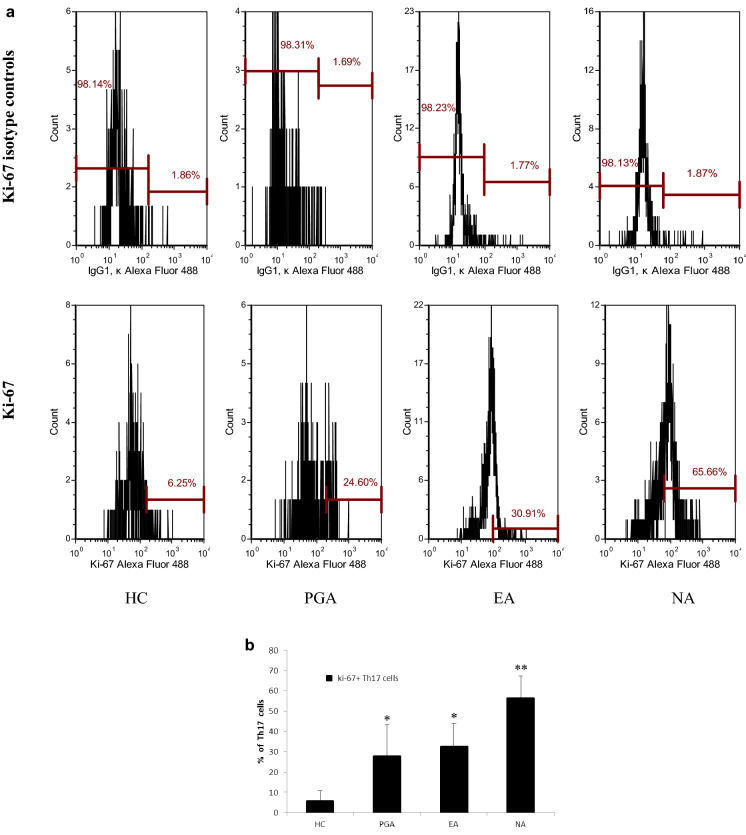
**a** Representative flow cytometric profiles of Ki-67^+^Th17 cells from asthmatics and healthy controls. **b** Percentage of Ki-67^+^Th17 cells in Th17 cells from asthmatics and healthy controls. *p < 0.01 versus healthy controls. **p < 0.01 versus PGA, EA and HC. *EA* eosinophilic asthma, *HC* healthy controls, *NA* neutrophilic asthma, *PGA* paucigranulocytic asthma

Representative flow cytometric profiles of Ki-67^+^Th17 cells from the samples are shown in Fig. 2a. Ki-67 positivity in peripheral Th17 cells was significantly higher in asthmatics compared to that in healthy controls (*p *< 0.01), in which Ki-67 positivity in peripheral Th17 cells was significantly higher in the NA group than those in the EA and PGA groups (*p *< 0.01) (Fig. 2b).

### Expression level of RORγt in PBMCs

**Fig. 3:**
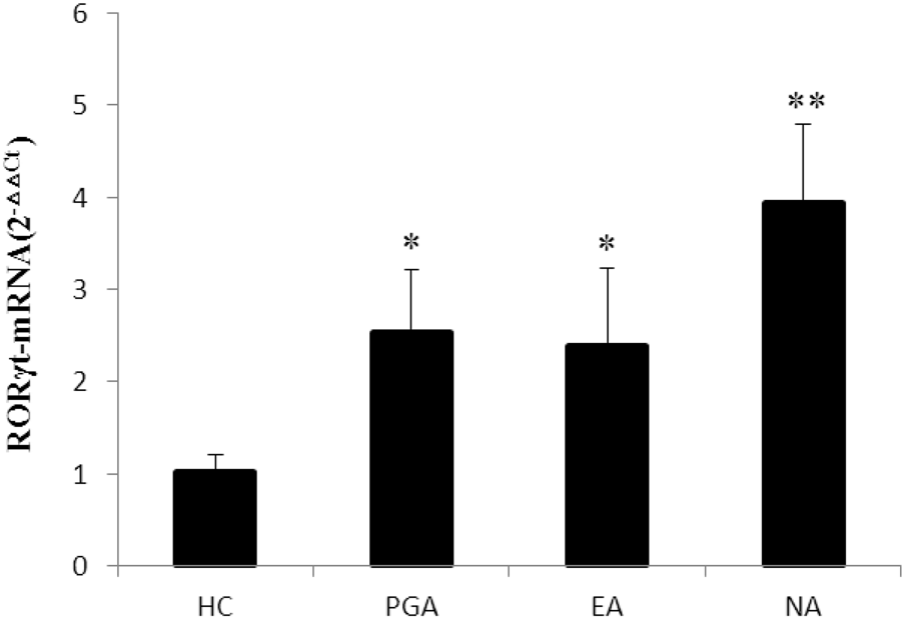
Expression level of RORγt in PBMCs from asthmatics and healthy controls. Using quantitative RT-PCR and 2^−△△Ct^ method, the data were presented as the fold change in RORγt expression normalized to an endogenous reference gene. *p < 0.01 versus healthy controls. **p < 0.01 versus PGA, EA and HC. *EA* eosinophilic asthma, *HC* healthy controls, *NA* neutrophilic asthma, *PGA* paucigranulocytic asthma

RORγt is considered to be the effector transcription factor which establishes the Th17 cells differentiation lineage. Quantitative RT-PCR and 2^−∆∆Ct^ method were used to compare the transcription level of RORγt in PBMCs between asthmatics and healthy controls. The datas were presented as the fold change in RORγt expression normalized to β-actin (an endogenous reference gene). The results revealed that the transcriptional level of RORγt in activated PBMCs was significantly higher in asthmatics than that in the healthy controls (*p *< 0.01), in which the transcriptional level of RORγt in PBMCs was significantly higher in the NA group than those in the EA and PGA groups (*p *< 0.01) (Fig. 3).

### Levels of IL-17, IL-8 and IL-5 in sputum

**Fig. 4:**
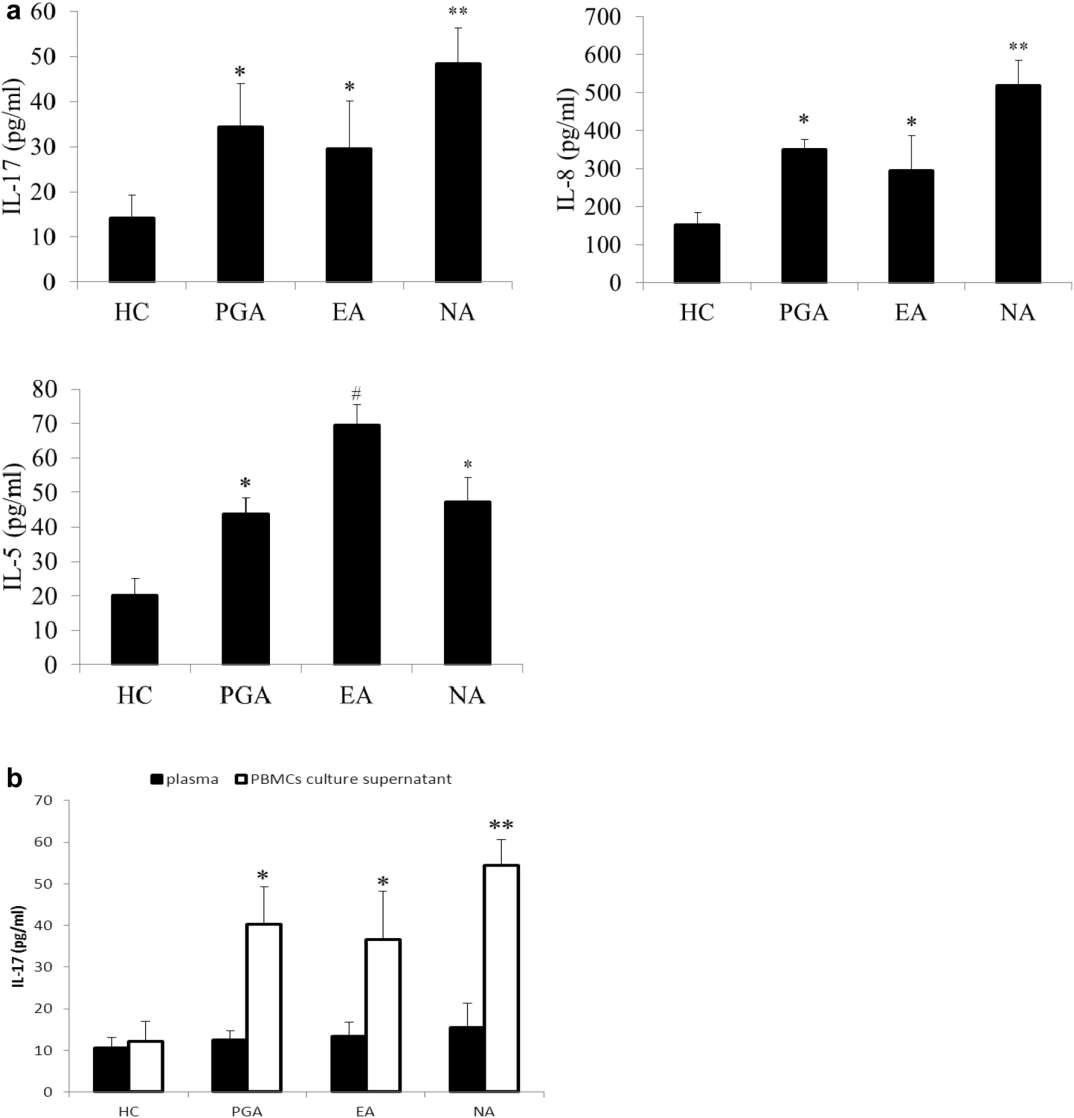
**a** Levels of IL-17, IL-8 and IL-5 in sputum from asthmatics and healthy controls. *p < 0.01 versus healthy controls. **p < 0.01 versus PGA, EA and HC. # *p *< 0.01 versus PGA, NA and HC. EA, eosinophilic asthma; HC, healthy controls; NA, neutrophilic asthma; PGA, paucigranulocytic asthma. **b** Levels of IL-17 in plasma and culture supernatant of activated PBMCs from asthmatics and healthy controls. *p < 0.01 versus healthy controls. **p < 0.01 versus PGA, EA and HC. *EA* eosinophilic asthma, *HC* healthy controls, *NA* neutrophilic asthma, *PGA* paucigranulocytic asthma

The concentrations of IL-17, IL-8 and IL-5 in sputum were measured by ELISA. The levels of IL-17, IL-8 and IL-5 in sputum were significantly increased in asthmatics compared to those in healthy controls (all *p *< 0.01), in which the levels of IL-17 and IL-8 in sputum were significantly higher in the NA group than those in the EA and PGA groups (*p *< 0.01), whereas the level of IL-5 in sputum was significantly higher in the EA group than those in the NA and PGA groups (*p *< 0.01) (Fig. 4a).

### Levels of IL-17 in plasma and culture supernatant of activated PBMCs

The concentrations of IL-17 in plasma and culture supernatant of PMA-stimulated PBMCs were measured by ELISA. The level of IL-17 in culture supernatant of activated PBMCs was significantly higher in asthmatics than that in healthy controls (p < 0.01), in which the level of IL-17 in culture supernatant of activated PBMCs was significantly higher in the NA group than those in the EA and PGA groups (*p *< 0.01). However, the level of IL-17 in plasma was not significantly increased in asthmatics compared to that in healthy controls (*p *> 0.05) (Fig. 4b).

### Correlations

**Fig. 5:**
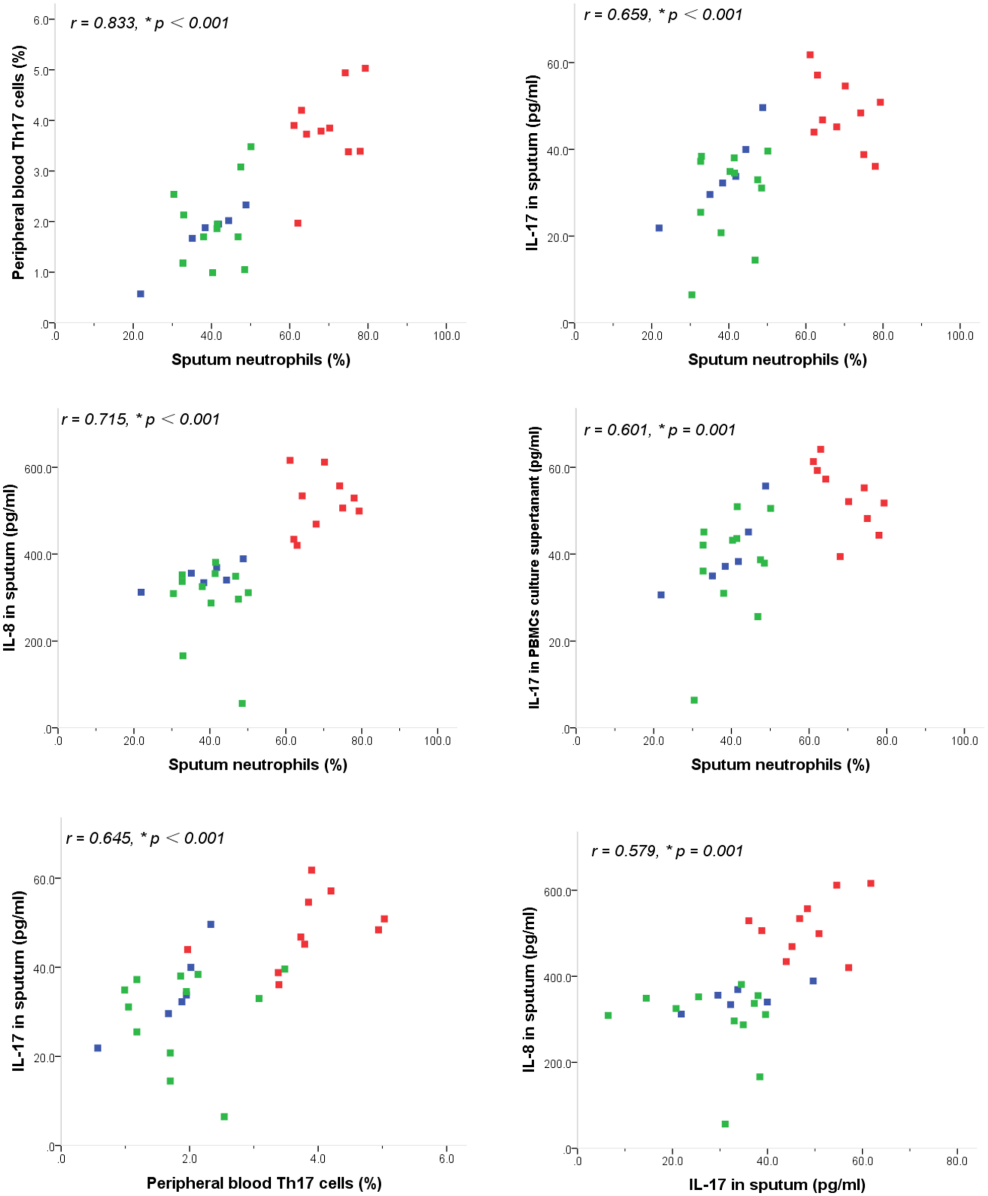

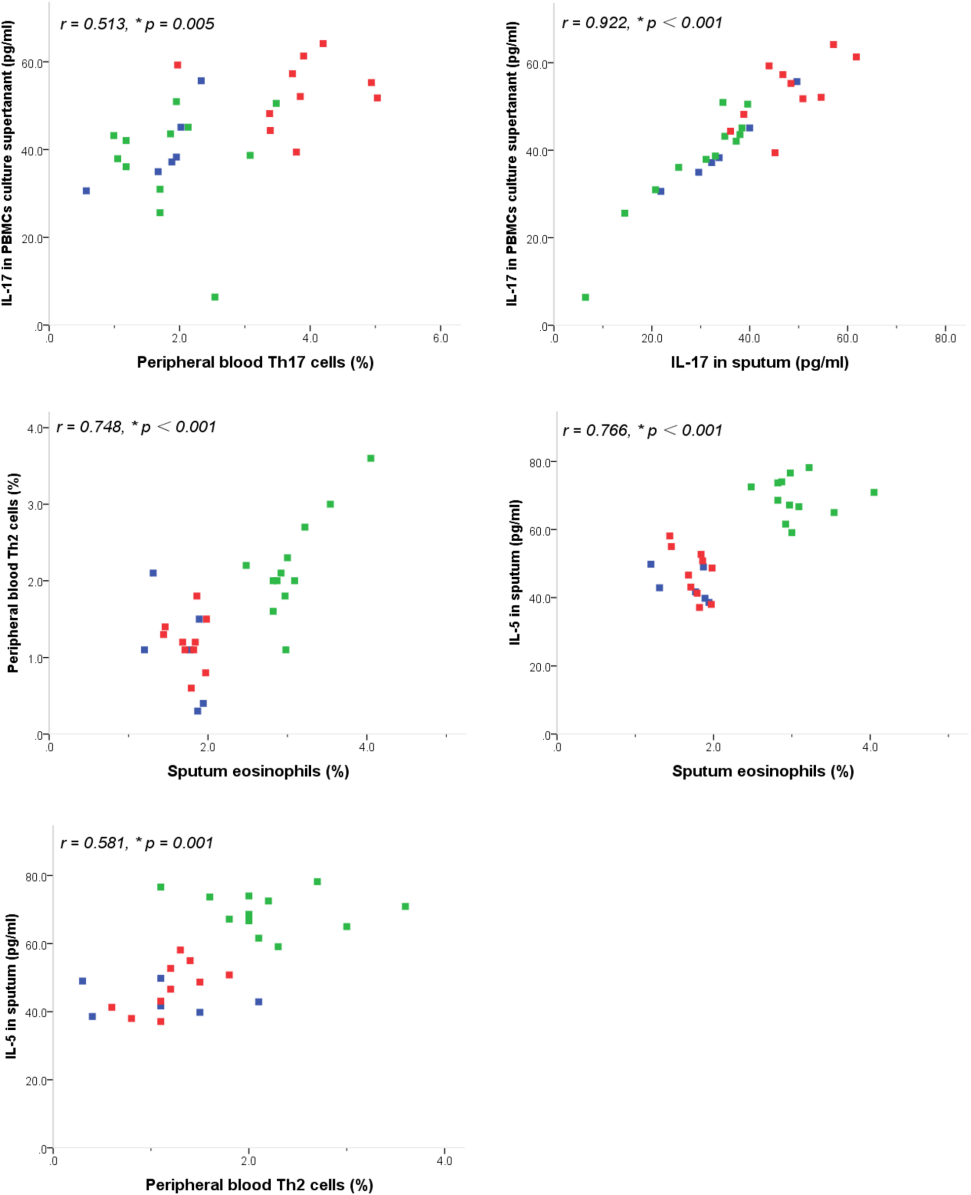
Correlations between sputum inflammatory cells and levels of peripheral blood Th17, Th2 cells and cytokine productions in sputum and in culture supernatants of activated PBMCs from asthmatics and healthy controls. *r* = Pearson’s correlation coefficients. *p < 0.01. *PGA* blue square, *EA* green square, *NA* red square

As shown in Fig. 5, the percentage of Th17 cells in peripheral Th cells and the concentrations of IL-17, IL-8 in sputum, as well as IL-17 in the culture supernatant of activated PBMCs were positively correlated with neutrophil level in sputum (*r* = 0.833, *p *< 0.001, *r* = 0.659, *p* < 0.001, *r* = 0.715, *p* < 0.001 and *r *= 0.601, *p *= 0.001, respectively) and with each other (*r* = 0.645, *p* < 0.001, *r *= 0.579, *p *= 0.001, *r* = 0.513, *p *= 0.005, and *r* = 0.922, *p* < 0.001, respectively). Moreover, the percentage of Th2 cells in peripheral Th cells and concentration of IL-5 in sputum were positively correlated with eosinophil levels in sputum (*r* = 0.748, *p *< 0.001 and *r* = 0.766, *p *< 0.001, respectively) and with each other (*r* = 0.581, *p *= 0.001).

## Discussion

Th17 cells represent a distinct lineage of CD4^+^Th cells, and mediate neutrophilic inflammation [10]. It’s well know that Th17 cells are characterized by the production of IL-17, IL-22 and IL-6 [17, 18]. Recent studies indicated that the eosinophilic/atopic phenotype of asthma driven by Th2 mechanisms was not the only immunologic pathway contributing to the pathogenesis of asthma [7, 8]. Th17 cells might potentially play an important role in the pathogenesis of asthma [11, 12]. Consistent with the recent study conducted by Zhao Y et al. [19], we found that the percentages of Th17 and Th2 cell in the peripheral Th cells, as well as the concentrations of IL-17 and IL-5 in sputum were higher in children with asthma than those in healthy controls, which suggested that both Th17-mediated immunity and Th2-mediated immunity were involved in the pathogenesis of childhood asthma. Whereas, the definitive role of Th17-mediated immunity in different inlfammatory phenotypes of asthma has not been fully established. To investigate this further, we classified the asthmatics as neutrophilic asthma, eosinophilic asthma, and paucigranulocytic asthma based on their phenotypes of airway inflammation, and found that the level of percentage of Th17 cells in peripheral Th cells, as well as the concentrations of IL-17 in sputum and the culture supernatant of activated PBMCs were significantly higher in children with neutrophilic asthma than those in children with eosinophilic and paucigranulocytic asthma, which indicated a predominance of Th17-mediated immunity in the pathogenesis of neutrophilic asthma. Recently, Zhao et al. have found that Th17-mediated immune response tended to increase with disease severity in the asthmatic patients [19]. In our study, we also found that FEV_1_ and PEF were significantly lower in the NA group than those in the EA and PGA groups, which might be partly explained by the predominance of Th17-mediated immune response in NA rather than in EA and PGA. Whereas, more studies need to be conducted to explore the relationship between the disease severity and phentype of airway inflammation in asthma. Also, it was observed that the percentage of Th2 cells in peripheral Th cells, and IL-5 in sputum were significantly higher in children with eosinophilic asthma than those in children with neutrophilic and paucigranulocytic asthma, which suggested that Th2-mediated immunity played a predominant role in the pathogenesis of eosinophilic asthma.

The of Th17 cells in neutrophilic inflammation depends on the ability of IL-17 to promote neutrophil production and chemotaxis via the induction of other proinflammatory cytokines and chemokines, such as IL-8, IL-6 and granulocyte colony-stimulating factor (G-CSF) [18]. Consistently, we observed that the concentrations of IL-8, IL-17 in sputum, and IL-17 in the culture supernatant of activated PBMCs, as well as the percentage of Th17 cells in the peripheral Th cells were all increased in the children with neutrophilic asthma and positively correlated with neutrophil levels in the sputum and with each other. Taken together, these results supported the concept that Th17-mediated immunity might be involved in the pathogenesis of neutrophilic asthma, and IL-17 might cause the airway neutrophilic inflammation by inducing other intermediate chemokines, such as IL-8. On the other hand, it is known that recruitment of Th2 cells that secrete IL-4, IL-5 and IL-13 accompanied by eosinophil recruitment to the airways has been considered integral to the pathogenesis of asthma [7–9]. Also, in this study, we observed that the percentage of Th2 cells in peripheral Th cells and IL-5 in sputum were increased in the children with eosinophilic asthma and positively correlated with eosinophil levels in the sputum and with each other. These results confirmed the predominant role of Th2-mediated immunity in the pathogenesis of eosinophilic asthma.

RORγt is an essential transcription factor, which plays a central role in differentiation of Th17 cells from naive CD4^+^ T cells, and is required for maintenance of IL-17 expression in differentiated Th17 cells in human [10]. We evaluated the differences of the RORγt mRNA expression in PBMCs among the asthmatics with different inlfammatory phenotypes. In parallel with increased level of peripheral blood Th17cells, the mRNA expression of RORγt in PBMCs was significantly higher in children with neutrophilic asthma than those in children with eosinophilic and paucigranulocytic asthma,which indicated a predominant role of Th17-mediated immunity in neutrophilic asthma at the transcriptional level.

The Ki-67 antigen is a nuclear antigen which is present only in proliferating cells, and is a useful and easy index for evaluating cell proliferative activity [20]. Also, we evaluated the differences of the expression of Ki-67 in the peripheral Th17cells among the asthmatics with different inlfammatory phenotypes in this study. In accordance with increased level of peripheral Th17cells, we observed parallel increased expression of Ki-67 in the peripheral Th17cells in children with neutrophilic asthma, which demonstrated a predominant role of Th17 cells proliferation in children with neutrophilic asthma. Further studies are necessary to better investigate the mechanisms underlying Th17 cells proliferation and survival, including apoptosis of Th17 cells in children with neutrophilic asthma.

Results from this study suggest that both Th17-mediated immunity and Th2-mediated immunity are involved in the pathogenesis of childhood asthma. The predominance of Th17-mediated immunity and Th17 cells proliferation was found in children with neutrophilic asthma. Th17 cells and IL-17 may mediate neutrophilic airway inflammation in asthma, indicating an important role for Th17-mediated immunity in the pathogenetic mechanisms as well as in the progression of childhood neutrophilic asthma. In addition, one of the limitations of this study was the small number of patients included in this study. The sample size shoule be expanded in the future.

## Data Availability

We would like to provide the raw data to support the information presented in this publication.

## Abbreviations

EA: Eosinophilic asthma
NEA: Non-eosinophilic asthma
NA: Neutrophilic asthma
PGA: Paucigranulocytic asthma
HC: Healthy control
RORγt: Retinoic acid-related orphan receptor γt
PBMC: Peripheral blood mononuclear cell
qRT-PCR: q Real-time quantitative reverse transcription-polymerase chain reaction
ELISA: Enzyme linked immunosorbent assay
Th cells: T helper cells
GINA: Global initiative for asthma
